# Isolated Rosai–Dorfman disease with craniocervical junction involvement in the foramen magnum: A case report

**DOI:** 10.1097/MD.0000000000017433

**Published:** 2019-10-04

**Authors:** Chao Wang, Yi Zou, Qingze Zeng, Hui Hong, Congkuan Zheng

**Affiliations:** a Department of Radiology; b Department of Pathology, The Second Affiliated Hospital, Zhejiang University School of Medicine, Hangzhou, Zhejiang; c Department of Radiology, Linhai Second People's Hospital, Taizhou, Zhejiang, China.

**Keywords:** craniocervical junction, foramen magnum, magnetic resonance imaging, Rosai–Dorfman disease

## Abstract

**Rationale:**

Rosai–Dorfman disease (RDD) is a rare benign histiocytic proliferative disease. RDD with cranio-spinal involvement in the foramen magnum is extremely rare. To the best of our knowledge, only 4 cases of RDD with craniocervical junction involvement have been reported so far. Herein, we present the fifth case of RDD with craniocervical junction.

**Patient concerns:**

A 26-year-old female presented with a sudden headache, accompanied by nausea and vomiting several times during the past half-month.

**Diagnoses:**

Magnetic resonance imaging (MRI) showed a well-defined, lobulated, homogenous mass in the left foramen magnum. The lesion was isointense on T1-weighted images (T1WI) and hypointense on T2-weighted images (T2WI), and showed homogeneously obvious enhancement following the intravenous administration of gadolinium. It was dural based and extending inferiorly along the spinal dura up to the cervical spinal canal. The brainstem was compressed and deflected to the right side. Initial diagnosis of meningioma with craniocervical junction involvement in the foramen magnum was made according to MRI findings. Final diagnosis of RDD was confirmed by histopathological and immunohistochemical examinations after subtotally surgical resection.

**Interventions:**

The bulk of lesion in the foramen magnum was removed surgically with suboccipital craniectomy because of brainstem compression.

**Outcomes:**

The patient recovered well and was discharged 17 days after the surgery.

**Lessons:**

RDD should be considered in patients with dural-based, extra-axial, well-circumscribed, hypo- to isointense on T1WI, hypo- to isointense on T2WI, enhancing intracranial or spinal lesions or both.

## 1 Introduction

Rosai–Dorfman disease (RDD), also known as sinus histiocytosis with massive lymphadenopathy, is a rare benign histiocytic proliferative disease. RDD can involve any nodal or extranodal site in all age groups. The extranodal manifestations that occur in 43% of all RDD cases most frequently affect the orbits, superior airway, bones, skin, gastrointestinal tract, genitourinary tract, endocrine glands, and central nervous system (CNS).^[1]^ CNS involvement is extremely rare in about 5% of cases.^[1]^ RDD with CNS involvement is usually shown as an extra-axial dura-based lesion and its most common locations are the convexity and the base of the skull.^[2]^ In this article, we present a case of RDD with extranodal involvement in the foramen magnum extending toward the cervical spinal canal in a 26-year-old woman. To the best of our knowledge, only 4 cases of RDD with craniocervical junction involvement have been reported since the disease was first described in 1969.^[2–5]^

## 2 Case presentation

This study was approved by the ethics review board of the Second Affiliated Hospital, Zhejiang University School of Medicine. Informed written consent was obtained from the patient for publication of this case report. A 26-year-old female had a sudden headache without obvious inducement, presented with persistent dull pain in the whole head, accompanied by nausea and vomiting several times in the past half-month. She was admitted at the local hospital and brain computed tomography (CT) was performed. Brain CT demonstrated a lesion in the foramen magnum. Pain medication was given orally for symptomatic treatment, and then the headache was relieved after medication. Two weeks later, she presented to our hospital for further treatment. On examination, her general examination was unremarkable. Review of systems did not reveal any abnormality. There was no lymphadenopathy on physical examination. Laboratory investigations were normal.

**Figure 1:**
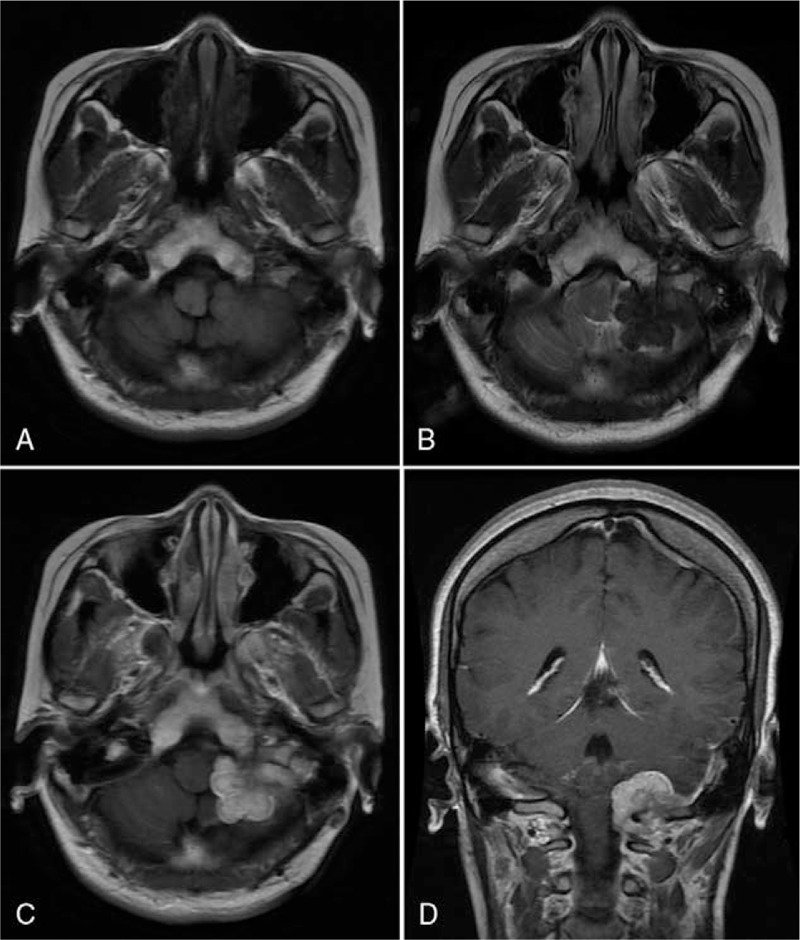
Contrast-enhanced magnetic resonance imaging (MRI) of the brain showed a well-defined, lobulated, homogenous mass in the left foramen magnum (A–D). The lesion was isointense on T1-weighted images (A), hypointense on T2-weighted images (B), and showed homogeneously obvious enhancement following the intravenous administration of gadolinium (C–D). It was dural based and extending inferiorly along the spinal dura up to the cervical spinal canal. The brain stem was compressed and deflected to the right side (A–D).

Contrast-enhanced magnetic resonance imaging (MRI) of the brain showed a well-defined, lobulated, homogenous mass in the foramen magnum on the left side. The lesion was isointense on T1-weighted images (T1WI) (Fig. 1A) and hypointense on T2-weighted images (T2WI) (Fig. 1B), and showed homogeneously obvious enhancement following the intravenous administration of gadolinium (Fig. 1C–D). It was dural based and extending inferiorly along the spinal dura up to the cervical spinal canal. The brain stem was compressed and deflected to the right side. The diagnosis of en plaque meningioma with cranio-spinal involvement in the foramen magnum was made before surgery.

**Figure 2:**
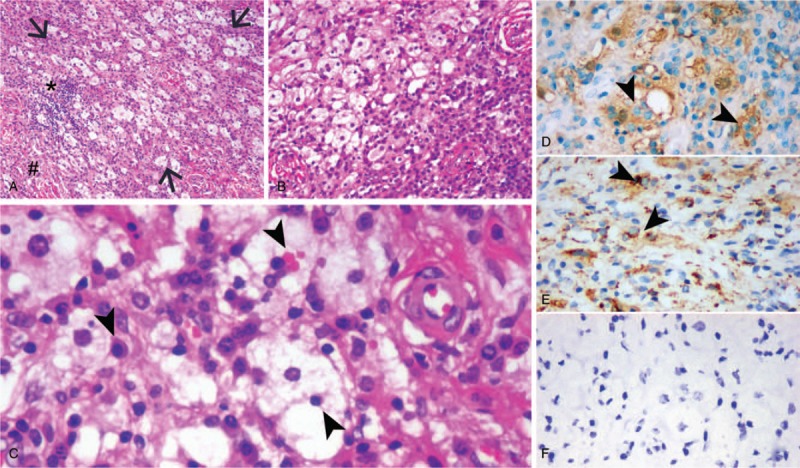
Hematoxylin and eosin staining histolopathological examination showed pale and large histocytes (arrow) aggregate intradural fibers (pound sign) accompanied with lymphocyte infiltrate (asterisk) (magnification, ×100) (A). Abundant plasma cells could be seen beside the histocyte mass (magnification, ×200) (B). Emperipolesis with histiocytic engulfment of intact plasma cells and red blood cells was conspicuous (arrowhead) (magnification, ×400) (C). Histocytes showed cytoplasmic and nuclear positivity for S-100, which made the engulfed lymphocytes and plasma cells more prominent (arrowhead) (D). The cytoplasm of the histocytes was immunoreactive for CD68 with stronger perinuclear positivity (arrowhead) (E). All the cells were immunohistochemically negative for CD1a (F).

**Figure 3:**
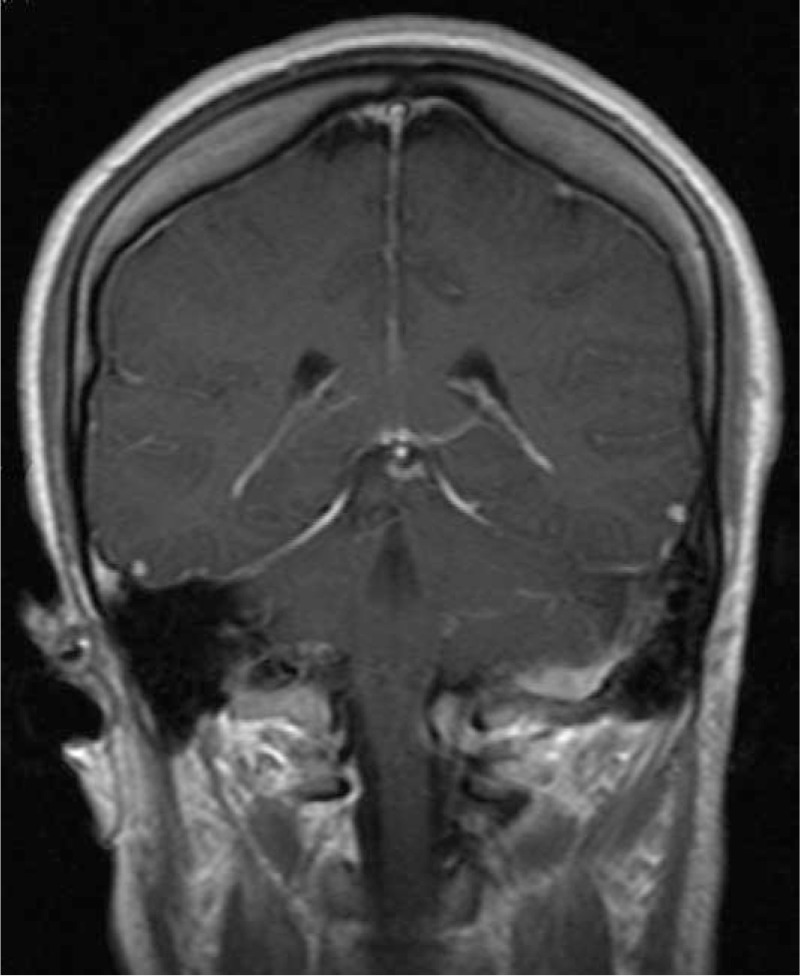
Postoperative MRI scan was performed on the 7th day after the operation revealed the bulk of lesion in the foramen magnum had been removed with little residual lesions.

The bulk of lesion in the foramen magnum was removed surgically with suboccipital craniectomy. Intraoperatively, the lesion was grayish white, firm attached to the dura. Histolopathological examination of the lesion revealed fibrous tissue with an infiltrate of inflammatory cells composed of histiocytes, lymphocytes, and plasma cells (Fig. 2A–C). The histiocytes contained abundant cytoplasm within intact lymphocytes (emperipolesis) (Fig. 2C). Immunohistochemical staining was positive for the markers S-100 (Fig. 2D) and CD68 (Fig. 2E), but negative for CD1a (Fig. 2F). The final pathological diagnosis of the lesion was RDD. Postoperative MRI scan performed on the 7th day after the operation revealed the bulk of lesion in the foramen magnum had been removed (Fig. 3). The patient recovered well and was discharged 17 days after the surgery. The patient was followed up by telephone after 5 months, and the patient was asymptomatic without disease progression.

## 3 Discussion

In 1969, 2 pathologists, Juan Rosai and Ronald Dorfman, described the disease and called it sinus histiocytosis with massive lymphadenopathy.^[6]^ RDD is a benign, non-neoplastic, self-limiting histiocytic disease. Its etiology and pathogenesis remain poorly understood. CNS involvement is rare, which only accounts for 5% of the cases with extranodal involvement.^[7]^ RDD in the CNS usually presents in the form of a well-defined, solitary, extraparenchymal supratentorial dura-based lesion. The clinical symptoms depend mainly on the location of the primary lesion, which include fever, headache, nausea and vomiting, dizziness, seizures, cranial nerve deficits, and weakness; these symptoms may be subacute, chronic, or recurrent.^[8,9]^ To the best of our knowledge, only 4 cases of RDD with craniocervical junction involvement have been previously reported so far (Table 1).^[2–5]^ We present the fifth case of RDD with craniocervical junction involvement in the foramen magnum. In all 5 cases of RDD with cranio-spinal involvement in Table 1, the most frequent neurological symptoms were motor deficits (2 cases) and headaches (2 cases) and neck pain (1 case).^[2–5]^

**Table 1:**
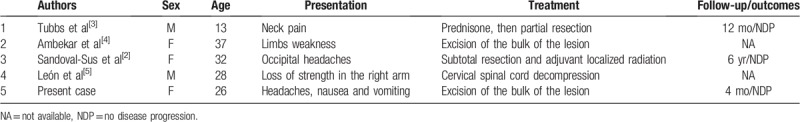
Summary of craniocervical Rosai–Dorfman disease cases previously reported in the literatures.

Although treatment approaches for RDD remain controversial, surgical resection is considered as the best treatment option for RDD in the CNS.^[2]^ Of these previously reported cases of craniocervical RDD in Table 1, all the 5 cases received subtotal resection but not total resection because of the extreme risk associated with the location of the lesion. In Table 1, case 1 received steroid medications before surgery for 6 weeks, but showed no change in the size. Although the disease is proven to be benign, there are individual differences in the prognosis of patients. Intracranial lesions regrowth or recurrence of symptoms occurred in about 14% of the cases with a mean follow-up period of 10.1 years.^[10]^ In Table 1, case 3 further received radiation after subtotal resection, but showed disease progression with an increase in the size 3 years later. Then, the patient was locally re-irradiated; the patient showed no disease progression 6 years after the initial diagnosis. Our case was followed up by telephone after 5 months, and was asymptomatic without disease progression. In addition, no instructions were given for postoperative follow-up for the case 2 and case 4 in Table 1.

MRI is currently the optimal diagnostic modality for evaluating lesions of intracranial RDD. Intracranial RDD most often shows a dural-based, extra-axial, well-circumscribed mass mimicking meningioma.^[11]^ The lesion commonly appears as iso- or hyperintense mass with clear borders relative to the peripheral brain parenchyma on T1WI; the lesion commonly appears as iso- or hypointense mass on T2WI. After injection of gadolinium contrast agent, the lesion shows homogeneously or inhomogeneously intense enhancement, and the dural tail sign can often be found.^[4,11]^ In our present case, brain MRI revealed a well-defined homogenous mass which is closely related to the dura mater in the foramen magnum extending toward the cervical spinal canal; the lesion was isointense on T1WI, hypointense on T2WI, and intensely enhanced with gadolinium. These above radiological findings are difficult to preoperatively distinguish RDD from meningioma. It should be noted that the T2 hypointensity is uncommonly seen in meningioma and should arouse the suspicion of a RDD lesion due to the presence of free radicals, focal necrosis, and fifibrosis.^[4]^ In addition, the absence of hyperostosis, bony erosion, or calcification should suggest RDD as the differential diagnosis of meningioma.^[11]^ Final diagnosis of RDD relies on pathological examinations, including histopathological and immunohistochemical examinations. Typically, the histiocytes contained abundant cytoplasm within intact lymphocytes which is called emperipolesis and histiocytes stain positive for S-100 and CD68, but negative for CD1a.^[12]^

## 4 Conclusion

Although RDD is rare and spinal cord compression is an unusual form of extranodal involvement, this disease should be considered in patients with dural-based, extra-axial, well-circumscribed, hypo- to isointense on T1WI, hypo- to isointense on T2WI, enhancing intracranial or spinal lesions or both. Treatment approaches for RDD remain controversial, but surgical resection is considered as the best treatment option for RDD in the CNS. Considering the extreme risk associated with the location of the foramen magnum with craniocervical junction involvement, subtotal resection should be advised to decompress cervical spinal cord. Final diagnosis of RDD should be confirmed by histopathological and immunohistochemical examinations.

## Author contributions

**Conceptualization:** Chao Wang, Congkuan Zheng.

**Data curation:** Chao Wang, Yi Zou, Qingze Zeng, Hui Hong.

**Investigation:** Qingze Zeng, Hui Hong.

**Resources:** Yi Zou.

**Writing – original draft:** Chao Wang.

**Writing – review & editing:** Chao Wang, Congkuan Zheng.
